# Knowledge, attitudes and practice of breast cancer screening among female health workers in a Nigerian urban city

**DOI:** 10.1186/1471-2407-9-203

**Published:** 2009-06-25

**Authors:** Adenike O Akhigbe, Vivian O Omuemu

**Affiliations:** 1 Department of Radiology, University of Benin Teaching Hospital, Benin City, Nigeria; 2 Department of Community Health, University of Benin Teaching Hospital, Benin City, Nigeria

## Abstract

**Background:**

Late presentation has been observed as the hallmark of breast cancer in Nigerian women and an earlier onset has been reported in this population. This study was designed to assess the awareness of female health workers about risk factors and screening methods for early detection of breast cancer.

**Methods:**

A cross-sectional descriptive study was carried out among female health workers in the two major government health institutions in Benin City, Edo State capital in Nigeria.

Data analysis was by SPSS version 10 and test of significance was done with differences considered significant at p < 0.05.

**Results:**

Three hundred and ninety-three (393) female health workers out of five hundred and five eligible subjects completed and returned the questionnaires, giving a response rate of 77.8%. One hundred and two (26%) were Doctors, two hundred and fifty-four (64.6%) Nurses, and thirty-seven (9.4%) were Radiographers, Laboratory Scientists and Pharmacists. A high proportion of our respondents had very poor knowledge about risk factors for breast cancer (55%). The awareness of mammography as a diagnostic method was very high (80.7%), but an extremely low knowledge of mammography as a screening method was found. Mammography practice of only 3.1% was found among those above 40 years of age who qualify for routine annual screening. Relatively low knowledge (45.5%) about Breast Self Examination (BSE) as a screening method was found.

**Conclusion:**

These female health workers who are expected to act as role models and educate the public had poor knowledge of risk factors for breast cancer and practice of breast cancer screening. There is very urgent need for regular update courses for health workers concerning breast cancer education including screening methods.

## Background

Breast cancer is one of the most dreaded conditions among women. Out of a list of 11 comparable conditions, 56% cited breast cancer as one of the top conditions they feared most [1].

Worldwide as well as in Nigeria, breast cancer has been reported as the most common cancer in women and the second leading cause of death [2,3].

It has been observed that breast cancer has a poorer outcome among African-American women compared with the whites due to more advanced stage at presentation [4] and this same trend has been reported among Nigerian women, as late presentation has been reported as the hallmark of breast cancer in this population [5-7]. On the other hand, early onset has been observed in this population compared with Caucasians.

If discovered early breast cancer can usually be cured; however, early detection through screening is the only way to reduce mortality [8].

Many studies have examined the role of health workers such as physicians [9] and nurses [10] in promoting breast cancer screening.

It has been shown that one of the strongest incentives for women to obtain a screening mammogram is the recommendation of their physician [9,11]. Even when female health workers are not directly involved in referring patients for breast cancer screening, they play an important role in creating an environment supportive of screening behaviors by offering positive role models.

Studies from developed countries show that attitude and orientation of healthcare providers are important determinants of use of breast screening program [12,13].

It has also been observed that for health workers to be effective as educators they must posses the appropriate knowledge, attitude and beliefs concerning the health behavior being promoted [8].

From various studies about breast cancer in Nigeria, very low level of knowledge about symptoms of breast cancer and screening methods has been reported [5,10,14].

Therefore there is a need for information and enlightenment, if patients are to present early in hospital.

Nurses, who form a major part of health workers, often give health talks in clinics and interact with patients and their relations; they can now play a crucial role in patient education about breast cancer screening methods [15].

In some parts of the developed world, the specialist breast-care nurse has evolved and these nurses are involved in public advocacy, care giving, support and research [16]. This is required more in developing countries such as Nigeria, where diagnostic facilities are inadequate. Apart from nurses, other health workers are also regarded as important role models in the communities were they live.

Various risk factors for breast cancer have been reported, and this include; increasing age, hormone replacement therapy (HRT), high dietary fat, excessive alcohol consumption, smoking and family history among others [4].

The importance of knowledge of these risk factors and the need for every woman to be aware of the need for surveillance on her breasts and the various ways to do this cannot be over emphasized.

The recognized screening methods include; breast self examination (BSE), clinical breast examination (CBE), and mammography. BSE as a screening method is controversial but it has been reported that this makes women more "breast aware", which in turn may lead to earlier diagnosis of breast cancer [17].

However, the practice of any of these screening methods is dependent on the awareness about breast cancer. If this knowledge is poor among those who should teach others, there will be difficulty promoting this life saving methods.

This study was designed to evaluate the knowledge, attitude and practice of breast cancer screening among female health workers in the two major government health institutions in Benin City, Edo state capital in Nigeria.

## Methods

This was a cross-sectional, descriptive study carried out between February-May 2006 among female healthcare workers in the two major government-owned health institutions in Benin-City, Edo State, Nigeria. The institutions used were the University of Benin Teaching Hospital, and the Central Hospital. The categories of female health workers included medical doctors, nurses, radiographers, laboratory scientists and pharmacists.

The minimum sample size required for the study was 111 based on mammography practice rate of 7.8% [10], using the formula for sample size determination for a cross-sectional descriptive study [18]. All the female doctors, radiographers, laboratory scientists and pharmacists employed by both hospitals at the time of the study were eligible to participate in the study. Only half of the nurses employed by both hospitals were included in the study. This was because of their relatively large number compared to the other cadres of health workers. The nurses in each department were selected by simple random sampling method (balloting). Only 393 out of a total of the 505 eligible female health workers completed and returned the questionnaires, giving a response rate of 77.8%.

A pre-tested, self-administered questionnaire was the tool for data collection. Informed consent was obtained from all respondents. Information was collected on socio-demographic characteristics, knowledge of breast cancer, its risk factors and screening methods as well as practice of breast self examination (BSE) and mammography.

### Scoring Scheme

Knowledge of risk factors was assessed by requesting the respondents to determine which of the following were risk factors for breast cancer – *increasing age, obesity, use of contraceptives, positive family history and breast feeding*. Thereafter, each correct response was scored one (1) point and each wrong response was scored zero (0). The total score ranged from 0–4. Respondents with scores 0–1 were considered to have poor knowledge, those with 2 points had a fair knowledge while those with 3–4 points had good knowledge. Mean score for knowledge of risk factors for each professional group was calculated and compared using one-way ANOVA.

#### For knowledge of mammography

The respondents were required to answer the following eight questions – *What is mammography, How often should mammography be done, its benefit, is it painful, is it safe, can it detect early stage breast cancer before it is palpable, what age group is most appropriate to start mammography screening and is mammography more beneficial in women 50 years and older than in those less than 50 years*. Each correct response was scored one (1) point and each wrong response was scored zero (0). The total score ranged from 0–8. Respondents with scores 0–3 were considered to have poor knowledge, those with 4–5 points had fair knowledge while those with 6–8 points had good knowledge. Mean score for knowledge of mammography for each professional group was calculated and compared using one-way ANOVA.

The practice of BSE was considered for each group of workers and compared with some selected demographic variables.

Data analysis was done using the SPSS version 10.0 statistical package and differences were considered significant at p < 0.05.

## Results

Table 1 shows the demographic profile of the respondents. A total of 393 female health workers with mean age 39.2 ± 9.9 years participated in the study. One hundred and two (26.0%) of them were doctors, 254 (64.6%) were nurses, 37 (9.4%) were radiographers, laboratory scientists and pharmacists. Most of the respondents were married 284 (72.3%) and Christians 382 (97.2%). One hundred and seventy-nine (45.5%) of the respondents had practiced for 1–10 years followed by 102 (26.0%) who had practiced for 11–20 years then 87 (22.1%) and 25 (6.4%) who had practiced for 21–30 years and greater than 30 years, respectively.

**Table 1:** Demographic characteristics of respondents

Characteristics	Frequency (N = 393)	Percent
**Age-group (years)**		

20–29	69	17.6

30–39	162	41.2

40–49	94	23.9

50–59	65	16.5

≥ 60	3	0.8

**Category of health workers**		

Doctors	102	26.0

Nurses	254	64.6

Radiographers/Laboratory scientist/pharmacist	37	9.4

**Marital status**		

Married	284	72.3

Not married	109	27.7

**Religion**		

Christian	382	97.2

Islam	10	2.5

African traditional religion	1	0.3

**Duration of practice (years)**		

1–10	179	45.5

11–20	102	26.0

21–30	87	22.1

> 30	25	6.4

Two hundred and fifty-two (63.6%) of the respondents knew that breast cancer was the most common cancer among women worldwide, while 271 (69.0%) knew that breast cancer was associated with a high incidence of death. The knowledge of specific risk factors for breast cancer is shown in Table 2. Majority of the total population 243 (61.8%) knew that a positive family history of breast cancer is a risk factor for the development of breast cancer followed by use of contraceptive 242 (61.6%), increasing age 96 (24.4%) and obesity 51 (13.0%). This same trend was noted specifically for the doctors, radiographers, laboratory scientists and pharmacists. For the nurses; majority (55.5%) knew that use of contraceptives is a risk factor for the development of breast cancer. The overall mean knowledge score was 1.61 ± 0.93 out of a maximum score of 4 points, (95 CI = 1.52 to 1.70). The doctors had a significantly higher mean knowledge score 2.32 ± 0.85 than the other categories of health workers, F = 51.047 p < 0.0001. It is important to note that a few of the respondents 4 (1.0%) believed that breast feeding is a risk factor for breast cancer.

**Table 2:** Knowledge of specific risk factors of breast cancer among respondents.

Parameters	Doctors *Freq %	Nurses *Freq %	Radiographers/Lab Sci/Pharmacist *Freq %	Total *Freq %
Family history	94 (92.2)	125 (49.2)	24 (64.9)	243 (61.8)

Contraceptives	82 (80.4)	141 (55.5)	19 (51.4)	242 (61.6)

Increasing age	39 (38.2)	48 (18.9)	9 (24.3)	96 (24.4)

Obesity	21 (20.6)	27 (10.6)	3 (8.1)	51 (13.0)

Breast feeding	1 (1.0)	2 (0.8)	1 (2.7)	4 (1.0)

Mean score (x ± sd)	2.32 ± 0.85	1.34 ± 0.83	1.46 ± 0.80	1.61 ± 0.93

95% CI	2.16 – 2.49	1.24 – 1.45	1.19 – 1.73	1.52 – 1.72

* Number and percentage responding correctly

Overall assessment of their knowledge revealed that 69 (17.5%) of them had good knowledge, 108 (27.5%) had fair knowledge while 216 (55.0%) had poor knowledge about the risk factors for breast cancer. The doctors (39.2%) had significantly higher knowledge of the risk factors than the nurses (9.8%) and the other categories of health workers (10.8%), X^2 ^= 94.056, df = 4, p < 0.0001. There was no consistent relationship between the knowledge of risk factors and the duration of practice

Three hundred and seventeen (80.7%) of the respondents were aware of mammography as a breast cancer diagnostic method rather than as a screening method. Other screening methods mentioned by them included breast self examination (BSE) or clinical breast examination (CBE) – 180 (45.8%), biopsy 39 (9.9%) and ultrasound scan 12 (3.1%). Their source of information about these screening methods include journals/books (31.1%), school (20.9%), hospital (14.8%), seminars (13.5%), electronic media (5.9%) and colleagues (4.1%).

Knowledge of specific aspects of mammography revealed that most of the respondents 301 (76.6%) knew that mammography can detect early stage breast cancer before it is palpable, while minority, 78 (19.8%) knew that mammography is more beneficial in women 50 years and older. This same trend was noted specifically for the doctors, radiographers, laboratory scientists and pharmacists but not for the nurses. The overall mean knowledge score was 3.74 ± 2.30 out of a maximum score of 8 points, (95 CI = 3.52 to 3.97). The doctors had a significantly higher mean knowledge score of 4.92 ± 1.77, compared with other categories of health workers, F = 19.752 p < 0.0001. (Table 3)

**Table 3:** Knowledge of mammography among respondents.

Parameters	Doctors *Freq %	Nurses *Freq %	+Others *Freq %	Total *Freq %
What is mammography?	76 (74.5)	144 (56.7)	17 (45.9)	237 (60.3)

How often should it be done?	49 (48.0)	83 (32.7)	8 (21.6)	140 (35.6)

What is its benefit?	86 (84.3)	132 (52.0)	24 (64.9)	242 (61.6)

Is mammography painful?	53 (52.0)	69 (27.2)	12 (32.4)	134 (34.1)

Is mammography safe?	86 (84.3)	124 (48.8)	18 (48.6)	228 (58.0)

Can it detect early stage breast cancer before it is palpable?	94 (92.2)	182 (71.7)	25 (67.6)	301 (76.6)

Age group most appropriate to start mammography screening.	39 (38.2)	51 (20.1)	15 (40.5)	105 (26.7)

Is mammography more beneficial in women ≥ 50 years than those < 50 years?	20 (19.6)	52 (20.5)	6 (16.2)	78 (19.8)

Mean score (x ± sd)	4.92 ± 1.77	3.33 ± 2.32	3.35 ± 2.36	3.74 ± 2.30

95% CI	4.57–5.27	3.04–3.61	2.56–4.14	3.52–3.97

* Number and percentage responding correctly.

+ Radiographers/Lab Sci/Pharmacist

Further assessment of their knowledge about mammography for screening revealed that 93 (23.7%) of the total population had good knowledge, while 159 (40.4%) and 141 (35.9%) respectively, had fair and poor knowledge of screening mammography. The doctors (40.2%) were significantly more knowledgeable about screening mammography than the other categories of health workers. X^2 ^= 35.943, df = 4, p < 0.0001. The knowledge about mammography increased significantly from16.8% in those who had practiced for 1–10 years to 44.0% among those who had practiced for more than 30 years, X^2 ^= 17.4 df = 6, p = 0.0079.

One hundred and sixty-two respondents making up 41.2% were above 40 years of age and qualify for screening mammography but only 5 (3.1%) of the respondents who are eligible for screening have ever had a mammogram.

Majority of the respondents 305 (77.6%) perform breast self examination (BSE). The respondents who practice BSE (1.65 ± 0.979) had a higher mean score for knowledge of risk factors of breast cancer than those who do not (1.47 ± 0.742) but the difference was not statistically significant, p = 0.1111. The respondents who practice BSE (3.96 ± 2.239) had a significantly higher mean score for knowledge of mammography than those who do not (2.98 ± 2.349), p = 0.0004.

Table 4 shows the comparison of BSE practice with selected demographic variables. The doctors had the highest proportion (81.4%) of those who practice BSE than the nurses (78.3%), and radiographers, laboratory scientists and pharmacists (62.2%), but the difference was not statistically significant, X^2 ^= 5.991, df = 2, p = 0.05.

**Table 4:** Comparison of BSE practice with selected demographic variables.

VARIABLE	PRACTICE		p-value
		
	Yes Freq %	No Freq %	
**Category of staff**			

Doctors	83 (81.4)	19 (18.6)	

Nurses	199 (78.3)	55 (21.7)	

Radiographers/Lab. scientists/Pharmacists	23 (62.2)	14 (37.8)	0.05

**Age-group (years)**			

20–29	45 (65.2)	24 (34.8)	

30–39	127 (78.4)	35 (21.6)	

40–49	71 (75.5)	23 (24.5)	

50–59	59 (90.8)	6 (9.2)	

≥ 60	3 (100.0)	0 (0.0)	0.0035*

**Duration of practice (years)**			

1–10	124 (69.3)	55 (30.7)	

11–20	87 (85.3)	15 (14.7)	

21–30	69 (79.3)	18 (20.7)	

> 30	25 (100.0)	0 (0.0)	0.0004*

**Total**	305 (77.6)	88 (22.4)	

*Statistically significant

Practice of BSE increased significantly with age from 65.2% among those who were 20–29 years to 100% in those who were 60 years and above, X^2 ^= 13.959, df = 3, p = 0.0035. Those who had practiced in their profession for more than 30 years (100%) had a significantly higher proportion of those who practice BSE than those who had practiced for less than 30 years, X^2 ^= 17.98, df = 3, p = 0.0004.

## Discussion

There have been many studies in Nigeria concerning clinical presentation of breast cancer among Nigerian women and late presentation has been observed in all the reports [19-21].

This late presentation is directly related to the level of awareness about breast cancer, the risk factors and practice of the screening methods among Nigerian women.

There has been reports about knowledge, attitude and practice of breast cancer screening methods among health and non-health workers in various parts of Nigeria [5,10,14,22].

Studies in developed countries show that attitude and orientation of healthcare providers are important determinants of use of breast cancer screening programs [12,13]

In order to function as effective promoter of breast cancer control through early detection, health workers must possess the relevant knowledge as well as appropriate attitude and belief concerning the disease and its early detection [8].

In this study, we found that majority of our respondents (55.0%) had very poor knowledge about the risk factors for breast cancer with an overall mean knowledge score of 1.61 ± 0.93.

This finding was unexpected, when compared with the report among Nurses in Lagos, Nigeria, where the nurses were found to be very knowledgeable about risk factors but lacked adequate knowledge about cancer risk estimation [10].

However this finding may be explained by the fact that apart from the doctors and nurses, radiographers, laboratory scientists and pharmacists were included in this study and these categories of health workers may not have adequate knowledge about the pathology of breast cancer as the doctors in their academic curricular or had close contact with the breast cancer patients like the nurses as part of routine care giving. If these categories of health workers are to be included as role models for creating awareness about breast cancer screening, an enlightenment program must be introduced as part of the general health maintenance knowledge for them.

As reported in the study in the Eastern Province of Saudi Arabia, there were misconceptions such as screening mammography increases the risk of breast cancer and majority of women develop breast cancer because of genetic link [23].

In this study, a misconception that breast feeding was a risk factor for breast cancer was observed. This misconception needs to be corrected, as breast feeding has been reported to confer some protection from breast cancer.

Awareness of mammography as a diagnostic method was very high (80.7%) but adequate knowledge was lacking (overall mean knowledge score was 3.74 ± 2.30) and only 23.7% of the respondents had good knowledge about the importance of screening mammography for early detection of beast cancer.

This is in contrast to findings among public health nurses in Singapore where the authors reported a high level of knowledge (96.1%) of screening mammography [24]. This very poor knowledge in our study may also be explained by the non-availability of the facility even in many of the government health institutions in the country including Benin City. However, the facility is available in some private centers within the city.

The knowledge about BSE as a screening method of about 45.8% was lower than that reported by Okobia et al [5] in their study among community-dwelling women within the same area with 87.2% score for knowledge about BSE as a screening method. This is rather worrisome for health workers who we expect to teach others in the community.

The practice of BSE was about 77,6% in this study population which is comparable to studies among Nigerian Nurses in Lagos from a general hospital (89%) and among registered Nurses with the Singapore Nursing Board (63%) [10,25].

An extremely low mammography practice of only 3.1% was found in this study among the 162 respondents who are above 40 years of age, which is lower than the report among Nurses in Lagos (7.8%) and an abysmally low rate compared with similar studies in Saudi Arabia (42.7%) and Singapore (35%) [10,23,25].

The fact that the facility is not readily available or accessible could be the reason for non-use. Even where it is available, the high cost of the procedure may be a barrier particularly for women in resource-poor setting like Nigeria.

There is yet no government-backed national breast cancer screening program in Nigeria, except few private initiatives. Considering the high incidence of breast cancer and the younger age at presentation as reported in various studies, [19-21] routine screening mammography has been proposed to start from the age of 40 years.

There was no international standardized questionnaire on breast cancer knowledge available and this may serve as a limitation in comparing our findings with other studies. The knowledge questions were based on available information from various studies and local myths about breast cancer in our environment.

## Conclusion

This study has revealed poor knowledge of breast cancer and the screening methods as well as low level of practice of breast cancer screening among these health workers. There is very urgent need for updating the various curricular of these categories of health workers to include courses in screening methods for early detection of such cancers like breast cancer and others like cervical, prostate and colorectal cancers. Regular update courses for health workers on health maintenance practices are also recommended.

Government hospitals will need to establish breast imaging units and it would be necessary to include screening mammography in the recently commenced national health insurance scheme as is practiced in developed countries.

The participation of non-governmental and charitable organizations in creating awareness about breast cancer will also be helpful in solving these problems.

## Competing interests

The authors declare that they have no competing interests.

## Authors' contributions

Both authors conceived of the study, performed the statistical analysis, interpreted the data and drafted the manuscript. Both authors read and approved the final manuscript.

## Pre-publication history

The pre-publication history for this paper can be accessed here:

http://www.biomedcentral.com/1471-2407/9/203/prepub
